# CDCA3 is a potential biomarker for glioma malignancy and targeted therapy

**DOI:** 10.1097/MD.0000000000038066

**Published:** 2024-05-10

**Authors:** Chengxi Han, Shuo Liu, Yunfeng Ji, Yuhua Hu, Jingwen Zhang

**Affiliations:** a Department of Neurosurgery, The Second Hospital of Hebei Medical University, Hebei, China.

**Keywords:** biomarker, CDCA3, cell cycle, checkpoint, glioma

## Abstract

CDCA3, a cell cycle regulator gene that plays a catalytic role in many tumors, was initially identified as a regulator of cell cycle progression, specifically facilitating the transition from the G2 phase to mitosis. However, its role in glioma remains unknown. In this study, bioinformatics analyses (TCGA, CGGA, Rembrandt) shed light on the upregulation and prognostic value of CDCA3 in gliomas. It can also be included in a column chart as a parameter predicting 3- and 5-year survival risk (C index = 0.86). According to Gene Set Enrichment Analysis and gene ontology analysis, the biological processes of CDCA3 are mainly concentrated in the biological activities related to cell cycle such as DNA replication and nuclear division. CDCA3 is closely associated with many classic glioma biomarkers (CDK4, CDK6), and inhibitors of CDK4 and CDK6 have been shown to be effective in tumor therapy. We have demonstrated that high expression of CDCA3 indicates a higher malignancy and poorer prognosis in gliomas.

## 1. Introduction

Gliomas are the most common primary central nervous system (CNS) tumors in adults, associated with high disability and mortality rates, leading to a very unfavorable prognosis. The World Health Organization (WHO) classifies gliomas into grades 1 to 4 based on malignant behavior.^[1]^ Despite the continuous improvement in surgical techniques, radiotherapy, and chemotherapy strategies, as well as the ongoing exploration of emerging treatments such as targeted therapy, immunotherapy, and tumor-treating fields (TTF), the prognosis for glioblastoma patients remains far from optimistic.^[2]^ Therefore, there is an urgent need for innovative treatment strategies for glioblastoma. The 2021 WHO classification of CNS tumors highlights that CDKN2A/B Homozygous Deletions exert a direct oncogenic impact by negating cell cycle inhibition and other concurrent mechanisms. They also serve as a molecular indicator that affects the classification and prognosis of IDH-mutant astrocytoma.^[3]^ This suggests that cell cycle-related therapy for glioma is important. One of the cell cycle-related genes, CDK4/6 inhibitor palbociclib, was the first drug acceleratedly approved by the US FDA for breast cancer treatment and has shown promising effectiveness in various mouse tumor models, including glioblastoma.^[4]^ However, despite the significant results observed in experiments with cell cycle inhibitors, their performance in clinical trials for glioblastoma has been suboptimal due to the lack of specific markers and cytotoxicity issues.^[5–9]^ Consequently, the quest for new cell cycle-related molecular markers for glioblastoma continues.

Cell division cycle-associated gene 3 (CDCA3), commonly referred to as trigger of mitotic entry 1 (TOME-1), was initially identified as a regulator of cell cycle progression, specifically facilitating the transition from the G2 phase to mitosis.^[10]^ Studies have shown that CDCA3 plays an important role in the development of various tumors.^[9]^ For example, CDCA3 has abnormally expressed in bladder urothelial carcinoma, non-small cell lung cancer (NSCLC), oral cancer, hepatocellular carcinoma and other tumors.^[11–14]^ At present, CDCA3 has been studied in the treatment of various cancers, but the research on glioma is still blank.

This study investigated the expression of CDCA3 in glioma and its prognostic significance. Additionally, we conducted an enrichment analysis to explore the mechanistic role of CDCA3 in glioma. Finally, by examining the relationship between CDCA3 and the glioma cell cycle checkpoint, we confirmed the role of CDCA3 in the cell cycle therapy of glioma.

## 2. Materials and methods

### 2.1. Data collection and processing

All clinical information, as well as bulk RNA-seq and array expression data, were acquired from Gliovis (http://gliovis.bioinfo.cnio.es/). The expression data for CDCA3 in a variety of cancer types and normal tissues was obtained from UALCAN (https://ualcan.path.uab.edu/). Prior to analysis, all the data underwent preprocessing through normalization. The subsequent data analysis was carried out using R 4.3 (R Core Team, 2023).

### 2.2. Bioinformatics analysis

The construction of the nomogram and calibration plots was accomplished using the RMS package within R software. Pearson correlation and correlograms were generated employing the circlize package and the corrgram package, respectively. To validate the biological processes, gene ontology (GO) analyses were performed using the R packages enrichplot and clusterProfiler. Gene Set Enrichment Analysis (GSEA) was conducted to compare the CDCA3 high expression group and low expression group, with the analysis tool available at https://www.gsea-msigdb.org/gsea/index.jsp. The significance of GSEA results was confirmed through the normalized enrichment score and false discovery rate (FDR). Pathways associated with genes having a *P* < .05 and FDR < 0.1 were visualized using Cytoscape version 3.8.2. Genes displaying a high correlation coefficient (*R* > 0.3 and *P* < .05) with CDCA3 were selected for heatmap presentation. Boxplots, Venn diagrams, and heatmaps were generated using the ggplot2, Venn diagrams, and pheatmap packages in R software.

### 2.3. Statistical analysis

R language version 4.3 was conducted for all statistical analyses. To evaluate differences in CDCA3 expression, Student *t* test was conducted. For survival analysis, we utilized the “Survival” and “survminer” packages in R. To dichotomize the continuous variables of CDCA3 expression, we determined the optimal cutoff values using the “surv_cutpoint” function from the “survminer” R package.^[15]^ Statistical significance was assessed using the log-rank test.^[16]^ Furthermore, we employed both univariate and multivariate COX proportional hazard models in R to calculate hazard ratios (HRs). Differences between groups were considered significant when *P* values were <.05.

### 2.4. Western blot

The glioma and normal brain tissues were minced by scissors and homogenized in RIPA lysis buffer with proteinase inhibitors, and the homogenate was centrifuged at 13,000 g, 4°C for 10 minutes, and the supernatant was collected. Cell protein was extracted using RIPA lysis buffer for 20 minutes at 4°C. Protein lysates (20 μg) were loaded and separated on SDS-PAGE, and the proteins were transferred to polyvinylidene difluoride (PVDF) membranes.^[17]^ Western blot was performed according to the above methods. β-Actin antibody was used as the internal reference, and the primary antibody was anti-CDCA3 antibody (1:10,000, Abcam). Next, use Imagej to analyze the data and GraphPad to plot the statistics.

## 3. Results

### 3.1. The overexpression of CDCA3 is correlated with the malignancy of gliomas

**Figure 1.**
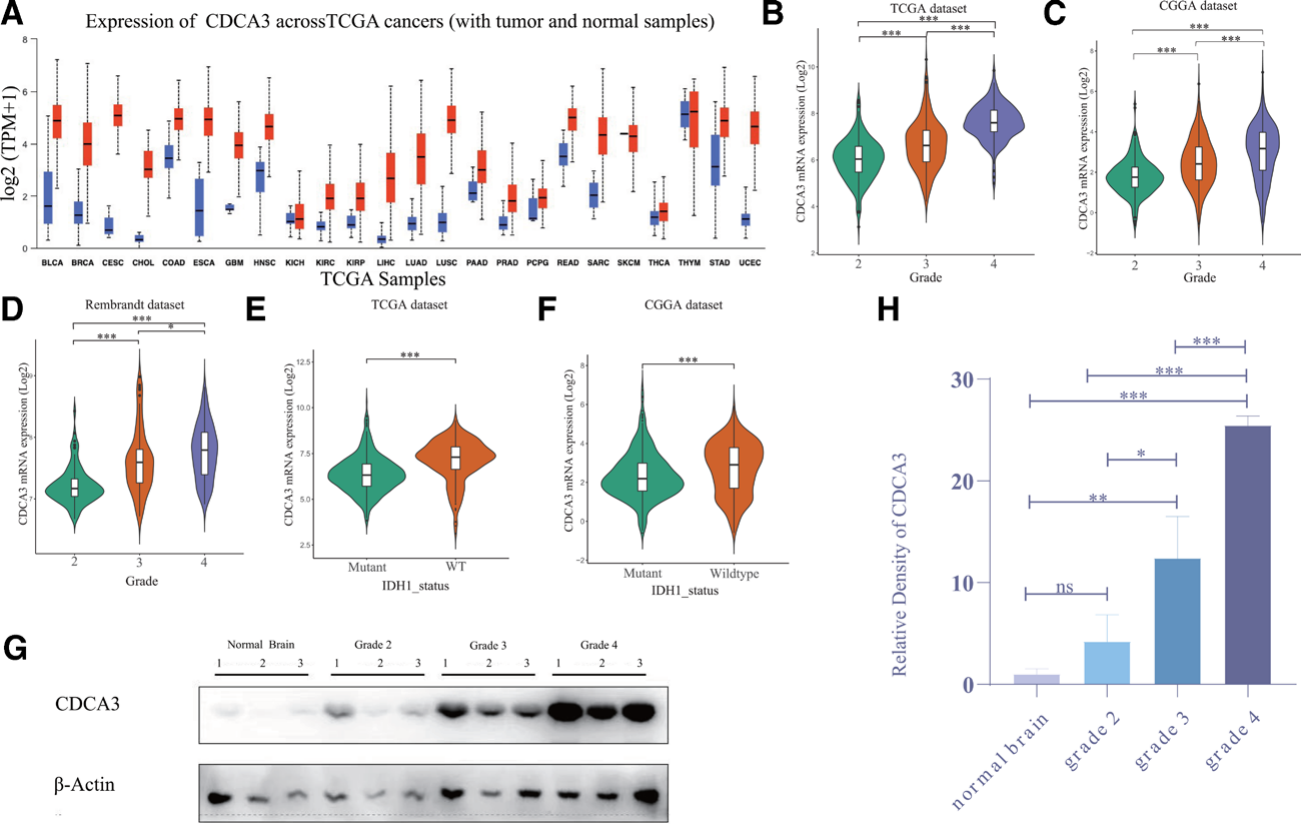
CDCA3 expression upregulated in glioblastoma (GBM). (A) The expression information for CDCA3 in tumor and normal tissues in multiple cancers in TCGA. Blue represents the normal tissue and red represents the tumor tissue. (B–D) CDCA3 expression level increase along with WHO grade in the TCGA database, CGGA database, Rembrandt database. (E) CDCA3 was upregulated in IDH1 WT group on TCGA database. (F) CDCA3 was upregulated in IDH1 Wild-type group-based CGGA database. (G) Western blot showed the expression of CDCA3 protein in normal brain tissue and WHO grade 2 to 4 glioma tissue. (H) According to Western blot gray-scale analysis, the expression of CDCA3 in normal brain tissue and LGG was significantly lower than that in high-grade gliomas. ^*^P < .05, ^**^P < .01, ^***^P < .001, and ^****^P < .001. NS = no significance.

First of all, CDCA3 exhibited elevated expression in various cancers, including gliomas, as illustrated in Figure 1A. Detailed analysis using data from the TCGA database unveiled a notably higher level of CDCA3 expression in glioblastoma (GBM) when compared to low-grade gliomas (LGG, grades 2 and 3), as depicted in Figure 1B. These findings were further substantiated by analyses of the CGGA and Rembrandt databases, as depicted in Figure 1C and D, respectively. Moreover, an in-depth examination of datasets from both TCGA and CGGA revealed that CDCA3 tends to be more prominently expressed in IDH wild-type gliomas. This heightened expression may potentially correlate with the heightened malignancy associated with this particular glioma subtype, as indicated by the statistical significance of *P* < .001 in Figure 1E and F. Western blot results of clinical samples showed that the content of CDCA3 in grade 4 gliomas was significantly higher than that in normal brain tissue and grade 2 and 3 gliomas (Fig. 1G and H), and in grade 3 gliomas, it is also higher than in normal brain tissue (*P* < .01, Fig. 1G and H).

### 3.2. CDCA3 was a potential prognostic indicator for glioma patients

**Figure 2.**
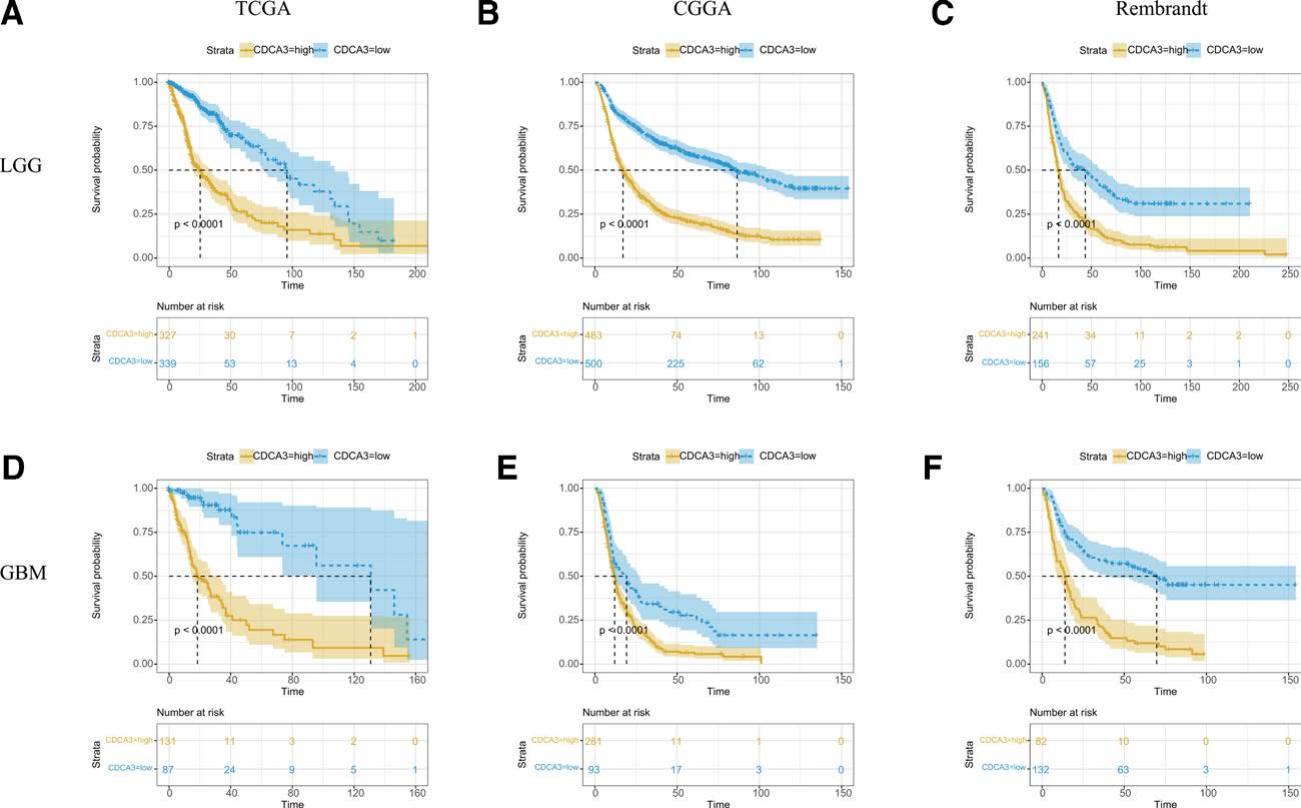
CDCA3 predicts poor prognosis of glioma patients. (A–C) LGG patients with increased expression of CDCA3 also get poor prognosis in TCGA dataset, CGGA database and Rembrandt dataset. (D–F) Higher CDCA3 expression portended poor prognosis for TCGA database, CGGA database and Rembrandt datasets.

To further assess the prognostic significance of CDCA3 in glioblastoma (GBM) and LGG, we conducted comprehensive analyses using data from the TCGA, CGGA, and Rembrandt datasets. Our findings indicated that upregulation of CDCA3 is a robust predictor of poor prognosis in glioma patients within the TCGA dataset (*P* < .0001, as illustrated in Figure 2A). Similarly, high CDCA3 expression is strongly associated with diminished overall survival (OS) in glioma patients, as evidenced by the CGGA and Rembrandt databases (both yielding *P* < .0001, as depicted in Figure 2B and C, respectively). The survival data for GBM corroborate these findings, consistently showing a significant association across TCGA, CGGA, and Rembrandt datasets (all with *P* < .0001, as shown in Figure 2D–F, respectively).

**Figure 3.**
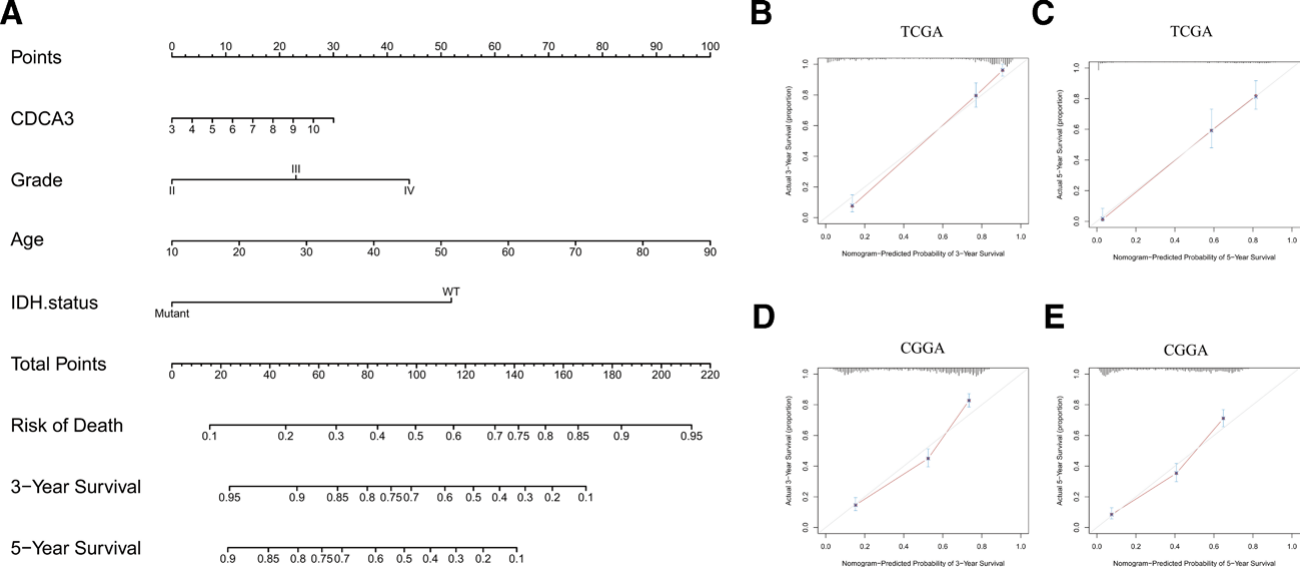
CDCA3-related prediction nomogram. (A) Nomogram for predicting 3- or 5-yr survival in glioma patients. The top row represents the point value for each variable. Rows 2 to 5 display the variables included in the nomogram. Each variable fits to point value based on glioma characteristics. The total points axis equals to the sun of the point value, and the lines downward to the total points are used to establish the liability of 3- or 5-yr survival. (B–C) The 3- or 5-yr survival prediction curve was obtained by COX regression analysis and TCGA database. (D–E) Calibration curves for predicting patient survival in CGGA dataset at 3 and 5 yr.

We conducted univariate and multivariate analyses using COX regression to identify factors associated with the prognosis of glioma patients, as summarized in Table 1. Based on the factors identified, we constructed a prognostic nomogram for predicting 3- and 5-year OS in glioma patients based on TCGA dataset (depicted in Figure 3A–C). To assess the predictive accuracy of this nomogram, we generated calibration plots for 3- and 5-year survival probabilities within an independent cohort sourced from CGGA (n = 1018). These plots revealed the closest alignment between the nomogram’s predictions and actual observations (Fig. 3D and E).

**Table 1:** Univariate and multivariate analysis of overall survival in the CGGA database.

Variables	Univariate analysis			Multivariate analysis		
	*P* value	HR	95% CI	*P* value	HR	95% CI
CDCA3	<2E−16	1.498	1.408–1.592	3.84E−10	1.255	1.169–1.347
AGE	2.95E−15	1.029	1.021–1.036	2.41E−02	1.008	1.001–1.015
WHO grade						
Grade 2	Reference			Reference		
Grade 3	2.99E−15	2.819	2.179–3.647	8.17E−11	2.449	1.869–3.208
Grade 4	<2E−16	7.935	6.184–10.182	<2E−16	4.413	3.277–5.942
IDH.status						
WT	<2E−16	3.098	2.615–3.669	2.61E−08	1.758	1.441–2.145
MU	Reference			Reference		

HRs = hazard ratios.

### 3.3. CDCA3 is involved in multiple regulatory mechanisms of gliomas, particularly in the cell cycle

**Figure 4.**
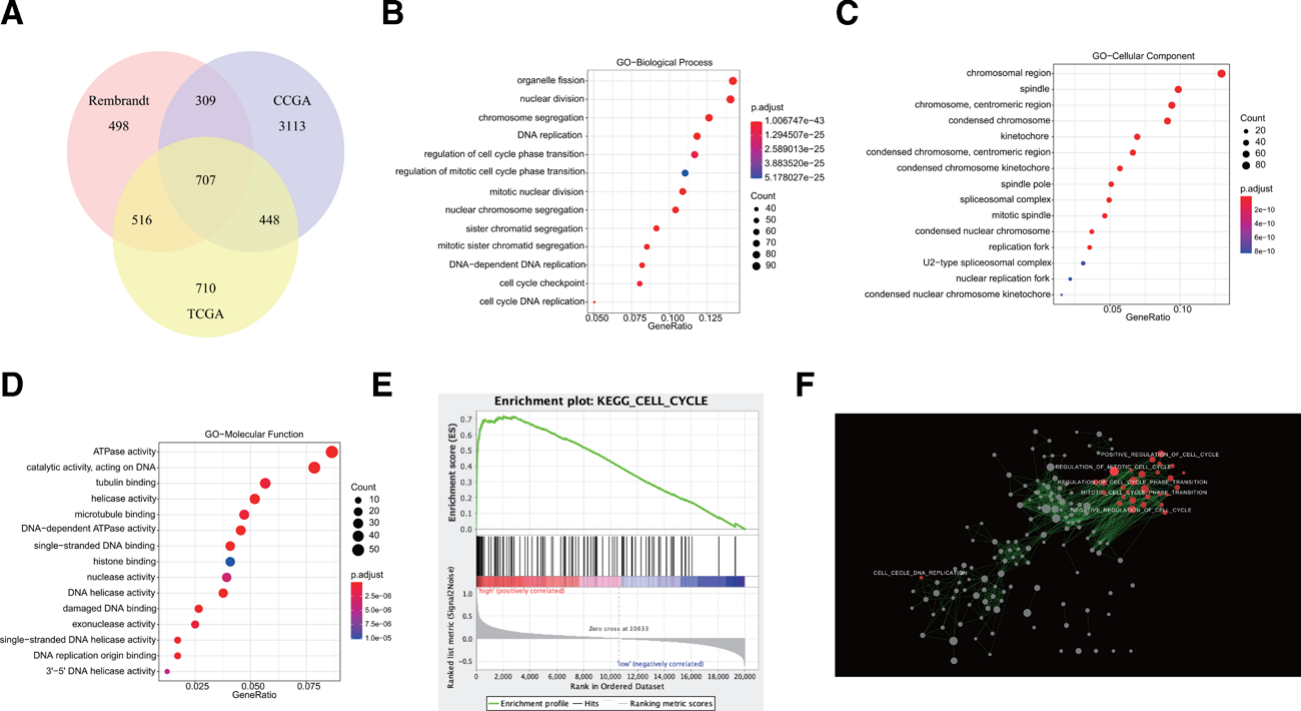
CDCA3 has a significant effect on glioma cell cycle. (A) The intersection of TCGA, CGGA, Rembrandt databases yielded 707 genes. (B) Gene Set Enrichment Analysis (GSEA) employed to verify the gene signatures: cell cycle. (C) The cystoscape of enrichment map results. Nodes represent gene sets, which were automatically arranged so that highly similar gene sets are placed close together, and node size represents the number of genes in the gene set. (D–F) The term cell cycle enriched most through gene ontology (GO) analysis on biological processes (BP), cellular component (CC), molecular function (MF).

In order to validate the involvement of CDCA3 in gliomas, we performed Pearson correlation tests independently within 3 distinct databases: TCGA, CGGA, and Rembrandt. Genes with correlation coefficients *R* > 0.4 (*P* < .05) were included to construct Venn diagrams (Fig. 4A). This process led to the identification of 707 genes that exhibited the highest correlation with CDCA3.

Subsequently, these 707 genes were subjected to GO analysis, which revealed that CDCA3 participates predominantly involved in biological processes associated with the cell cycle, such as organelle fission, nuclear division, chromosome segregation, DNA replication, regulation of cell cycle phase transition, and so on (Fig. 4B). Furthermore, the localization of CDCA3 within the cell, including the chromosomal region, spindle, centromeric region of chromosomes, and condensed chromosomes (Fig. 4C), also correlates with cell cycle-related functions. Moreover, CDCA3’s molecular functions in gliomas primarily encompass ATPase activity, tubulin binding, helicase activity, microtubule binding, and similar activities (as shown in Figure 4D).

Simultaneously, the enrichment of CDCA3 in the glioma cell cycle was validated through GSEA (Fig. 4E). Then, we visualized the molecular mechanisms associated with CDCA3 using Cytoscape, where each node represents a distinct signaling pathway, and the node size corresponds to the number of genes within that pathway. Notably, nodes related to the cell cycle are highlighted in red. This visualization clearly demonstrates the significant role of CDCA3 within the glioma cell cycle (Fig. 4F).

### 3.4. CDCA3 plays a crucial role in the cell cycle therapy for gliomas

**Figure 5.**
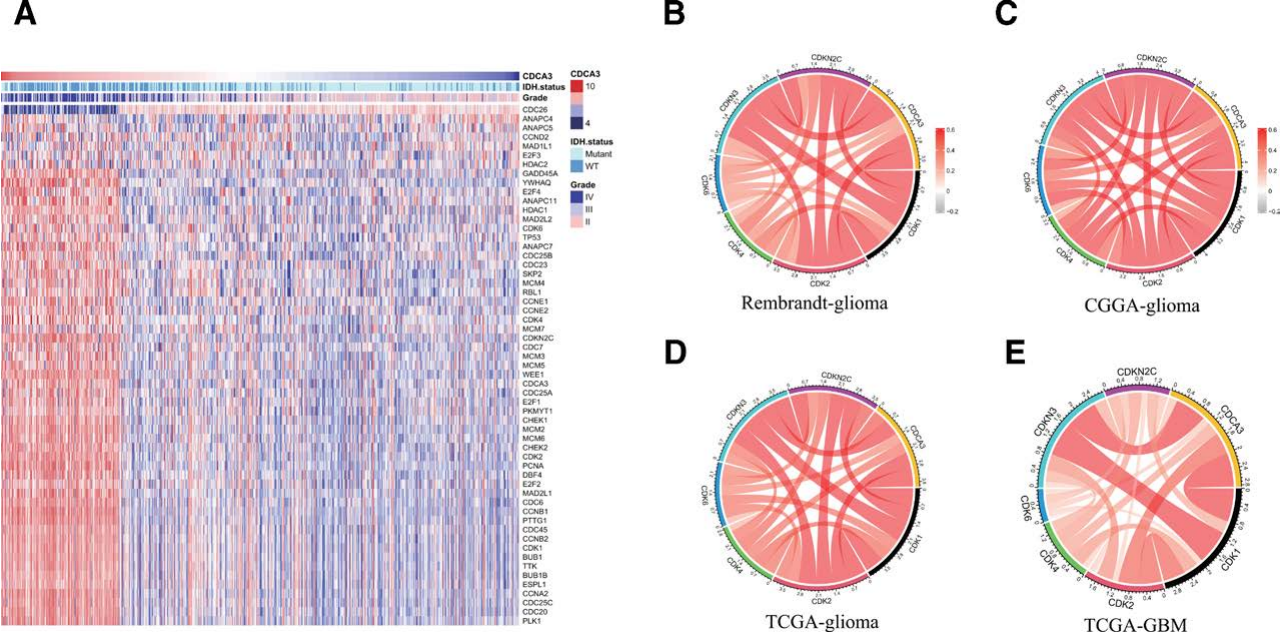
CDCA3 co-expression gene. (A) Heatmap based on TCGA dataset visualizing the relationship between CDCA3 and more than 50 cell cycle-related genes. (B–E) The associations between DDX60 and cell cycle-related genes including CDK1, CDK2, CDK4, CDK6, CDKN3, and CDKN2C based on TCGA, Rembrandt dan CGGA datasets were presented. CDKN = cyclin-dependent kinase.

To further validate the role of CDCA3 in the glioma cell cycle, we generated a heatmap illustrating the expression of cell cycle-related genes, indicating a positive correlation between CDCA3 and most of these genes in gliomas (Fig. 5A). Subsequently, we employed data from the TCGA, CGGA, and Rembrandt databases to create circos plots based on the correlation of CDCA3 with common cell cycle checkpoint genes such as CDK1, CDK4, and CDK6 (Fig. 5B–E). Additionally, we constructed a circos plot illustrating the correlation of CDCA3 with cell cycle checkpoint genes in GBM using the TCGA database. These findings demonstrate a strong association between CDCA3 and cell cycle checkpoint genes. These findings not only suggested that CDCA3 could serve as an indicator for assessing the efficacy of cell cycle therapy in gliomas but also highlights its potential as a novel target for cell cycle-based glioma treatment.

## 4. Discussion

Despite the ongoing advancements in glioma treatments, such as molecular targeted therapy, immunotherapy, and TTF, the prognosis for glioma patients remains unfavorable. There is a continual need for exploration of new treatment targets in gliomas. This study has identified and validated a novel molecular target, CDCA3, for glioma molecular therapy. Firstly, we demonstrated the high expression of CDCA3 in gliomas, and its expression level showed a positive correlation with the malignancy of gliomas. We verified the results of our bioinformatic analysis through Western blot experiment, confirming that CDCA3 gene is highly expressed in GBM, thus affecting the prognosis of glioma patients. Secondly, through survival analysis, we observed that both glioma and GBM patients with high CDCA3 expression had a poorer prognosis. We also constructed a nomogram to predict the 3- and 5-year prognosis of glioma patients. Furthermore, various methods including GO and GSEA analyses revealed that CDCA3 primarily participates in cell cycle-related functions in gliomas. Finally, this study suggests that CDCA3 could potentially serve as a biomarker for cell cycle-based therapy in gliomas.

CDCA3, also known as TOM-1, is located on human chromosome 12p13. The gene is 7271bp long and contains 6 exons.^[8]^ This protein contains 286 encoded amino acids and contributes to human physiological and pathological processes by regulating various downstream cytokines. There has been evidence of increased CDCA3 expression in many cancer types, such as bladder tumor, NSCLC, oral cancer, as well as hepatocellular carcinoma (HCC).^[11–14]^ In this study, we found that the upregulation of CDCA3 expression was positively correlated with the grade of glioma, and that CDCA3 expression was upregulated in gliomas with MGMT unmethylation and IDH wild-type. MGMT promoter methylation was initially identified as a prognostic and predictive marker within the diagnosis of GBM in patients treated with temozolomide.^[18]^ IDH is an independent factor indicating the prognosis of glioma patients.^[19]^ Therefore, these results might suggest that glioma patients with high CDCA3 expression are not sensitive to temozolomide and have a poor prognosis.

Previous studies have shown that CDCA3 plays an important role in promoting the development of ovarian cancer. As expected, our study showed that CDCA3 is closely associated with poor prognosis in both GBM and LGG. Nomograms are widely used in cancer prognosis primarily because of their ability to reduce statistical predictive models to a single numerical estimate of the probability of an event, such as death or recurrence, tailored to an individual patient’s profile.^[20]^ Therefore, we set CDCA3 as a prognostic indicator of glioma and established a nomogram with a risk classification system. The 4 parameters in this nomogram are consistent with clinical relevance and COX regression analysis.^[21]^ Studies have shown that gender, age, WHO grade and IDH status are related to the prognosis of glioma.^[22–24]^ However, after we conducted univariate and multivariate COX analysis, we did not find a correlation between gender and glioma prognosis. So we exclude gender from the observable range of the nomogram. The calibration plots of the 2 external validation cohorts were highly fit, indicating that the nomogram performed well in predicting 3- or 5-year survival in glioma patients.

In most adult mammalian cells, the cell cycle is tightly regulated through multiple molecular pathways and checkpoints. However, cell cycle dysregulation appears to be the primary mechanism underlying the immortal proliferation of malignant glioma cells.^[25–27]^ Our research shows that CDCA3 is enriched in cell cycle DNA replication, regulation of cell cycle phase transition, regulation of mitotic cell cycle phase transition, and cell cycle checkpoint. This indicates that CDCA3 plays an important role in the glioma cell cycle.

Finally, we visualized the close association between CDCA3 and common glioma cell cycle checkpoint markers, including CDK6, CDK4, CDK2, CDK1, CDKN3, and CDKN2C, using circos plots. A literature review shows that cyclin D1 forms a complex by combining with cyclin-dependent kinase CDK4/6 to promote the transition of the cell cycle from G0/G1 phase to S phase.^[23]^ Three small-molecule CDK4/6-inhibitors have been extensively characterized in preclinical studies: palbociclib and ribociclib, which are highly specific CDK4/6-inhibitors, and abemaciclib, which inhibits CDK4/6 and other kinases.^[28]^ For example, CDK4/CDK6 inhibitor palbociclib reduces tumor growth by reducing retinoblastoma (RB) protein phosphorylation and cell cycle arrest, which induces G1/S phase transition.^[29]^ In recent years, CDK4/CDK6 inhibitors have become a powerful drug for the treatment of glioma. Cyclin-dependent kinase CDK2 is a serine/threonine protein kinase that inhibits CDK2 activity by inducing P21 after DNA damage and plays a key role in the G1/S transition.^[30,31]^ Cyclin-dependent kinase inhibitor 2C (CDKN2C) protein encoded by its eponymous gene (CDKN2C), it is a member of the INK4 family. The CDKN2C protein can bind to CDK4 or CDK6 and reduce CDK kinase activation, contributing to cell cycle arrest in the G phase.^[32]^ In addition, studies have shown CDKN3 protein was expressed at low levels in G0/1 and S phase and was increased in M phase in parallel with phosphorylation of histone H3 Ser-10, which is a marker of M phase.^[33]^ In our study, we found that CDCA3 is positively correlated with the expression of CDKN3, CDKN2C, CDK6, CDK4, CDK2, and CDK1 in gliomas. These findings indicate that CDCA3 not only would serve as a predictor for the efficacy of glioma cell cycle therapy, but might also become a novel biomarker for cell cycle-based glioma treatment. The limitation of this paper is that there is no cytological experiment, only bioinformatics analysis.

In summary, this study has demonstrated that high expression of CDCA3 indicates a higher malignancy and poorer prognosis in gliomas, with its mechanism closely linked to the cell cycle. Our study can introduce new diagnostic and therapeutic targets for glioma, which may change the prognosis and survival time of GBM patients. However, our study is still limited by the lack of cytological experiments to fully verify the relationship between CDCA3 and GBM cell cycle, which needs further research.

## Acknowledgments

We would like to thank the reviewers and editor for their valuable comments.

## Author contributions

**Conceptualization:** Shuo Liu.

**Formal analysis:** Yunfeng Ji.

**Methodology:** Yuhua Hu.

**Supervision:** Jingwen Zhang.

**Validation:** Chengxi Han.

**Visualization:** Chengxi Han.

**Writing – original draft:** Chengxi Han.

**Writing – review & editing:** Jingwen Zhang.
